# Responding to the Return of Influenza in the United States by Applying Centers for Disease Control and Prevention Surveillance, Analysis, and Modeling to Inform Understanding of Seasonal Influenza

**DOI:** 10.2196/54340

**Published:** 2024-04-08

**Authors:** Rebecca K Borchering, Matthew Biggerstaff, Lynnette Brammer, Alicia Budd, Shikha Garg, Alicia M Fry, A Danielle Iuliano, Carrie Reed

**Affiliations:** 1 National Center for Immunization and Respiratory Diseases Centers for Disease Control and Prevention Atlanta, GA United States; 2 Fulton County Board of Health Atlanta, GA United States

**Keywords:** disease burden, modeling, seasonal influenza, surveillance

## Abstract

We reviewed the tools that have been developed to characterize and communicate seasonal influenza activity in the United States. Here we focus on systematic surveillance and applied analytics, including seasonal burden and disease severity estimation, short-term forecasting, and longer-term modeling efforts. For each set of activities, we describe the challenges and opportunities that have arisen because of the COVID-19 pandemic. In conclusion, we highlight how collaboration and communication have been and will continue to be key components of reliable and actionable influenza monitoring, forecasting, and modeling activities.

## Context

The COVID-19 pandemic disrupted the typical timing, intensity, and duration of influenza virus activity in the United States and many parts of the world [1,2]. These disruptions are likely due in part to the adoption of nonpharmaceutical interventions, such as mask wearing and physical distancing, which were deployed to prevent SARS-CoV-2 transmission and likely also reduced transmission of influenza viruses and other respiratory viruses [1]. Reductions in the use of nonpharmaceutical interventions in the United States during the 2022-2023 influenza season compared to what was observed during the 2020-2021 and 2021-2022 seasons may have contributed to a return of influenza virus activity more comparable to levels observed during some prepandemic seasons, which had annual estimates of 9 million-41 million illnesses, 140,000-710,000 hospitalizations, and 12,000-52,000 deaths between 2010 and 2020 [3]. The interim estimated burden of influenza for the 2022-2023 influenza season indicated that between 27 and 54 million illnesses, 300,000 and 650,000 hospitalizations, and 19,000 and 58,000 deaths occurred that season [4]. The 2022 and ongoing 2023 influenza seasons observed in the Southern Hemisphere winter (approximately May to September) were characterized by the circulation of multiple seasonal influenza viruses (ie, influenza A/H3, A/H1, and B Victoria) [5]; atypical timing patterns, including early and late seasons; or increased intensity compared to recent seasons during the COVID-19 pandemic. However, changes in care-seeking behaviors, testing practices, and general reporting practices due to the pandemic complicate the interpretation of influenza surveillance data.

The experience of the Southern Hemisphere in 2022 and 2023 [5] and the atypically early and rapid start to the 2022-2023 influenza season in the United States [6-8] serve as reminders that seasonal influenza remains a major public health threat. Influenza burden during the 2022-23 season was elevated compared to activity observed in the United States during the first 2 years of the COVID-19 pandemic [4,9], and early and high rates of influenza-associated hospitalizations were observed, particularly in children [7,8,10]. Over the past decade, the Centers for Disease Control and Prevention’s (CDC’s) Influenza Division has worked internally and with external collaborators to develop, validate, and improve forecasting, mathematical modeling, and analytical tools that help translate US influenza surveillance data into action by forecasting short-term influenza activity and characterizing the burden and severity within and across influenza seasons. With the decreased influenza activity observed in the 2020-2021 season, atypical activity and timing in the US 2021-2022 and 2022-2023 seasons, and the Southern Hemisphere’s 2022 and 2023 seasons, continued circulation of SARS-CoV-2, and the return of other respiratory pathogens (eg, respiratory syncytial virus [RSV]) [1,11], we summarize these activities to highlight how these tools can help inform influenza prevention and control efforts during future seasons in the postpandemic respiratory virus landscape.

## Data From Surveillance Systems Provide the Foundation for Influenza Situational Awareness and Risk Communication

Multiple surveillance systems provide complementary information to create a comprehensive picture of influenza activity in the United States [6,12]. These information streams are fundamental for situational awareness, risk communication, and subsequent modeling and analysis. Types of surveillance data collated and distributed by the CDC include influenza hospitalizations (hospitalization rates by age, sex, and race or ethnicity, as well as clinical characteristics and outcomes) [9,13]; influenza-related mortality (the National Center for Health Statistics Mortality Surveillance Data and pediatric influenza-associated mortality); virologic (eg, percent positivity, influenza virus type or subtype, and genomic characterization of influenza viruses, which can be used to assess the match of circulating strains to vaccine components); and syndromic outpatient illness [12]. Maintaining these data sources requires coordination within the CDC as well as with international organizations, vital statistics offices, state, and local public health partners in both clinical and laboratory settings, hospitals, and health care providers.

Weekly FluView [6] reports provide systematic data updates and interpretation from the many influenza surveillance systems. These reports, related data updates, and visualizations provide a valuable resource for the CDC, external partners, and the public to understand and interpret influenza activity. Regular and rapid dissemination of surveillance data that is easily accessible and interpretable plays an important role in risk communication and promotion of prevention and mitigation measures, such as vaccination, testing, antiviral use, and nonpharmaceutical interventions.

Given the cocirculation of multiple respiratory viruses with the potential to cause severe illness, interpreting influenza virus data alongside complementary surveillance data for other respiratory viruses provides additional context to the added burden of influenza on the population and the health care system. Symptom-based surveillance for influenza has traditionally been complicated, due in part to overlapping symptomologies. Accordingly, data from the US outpatient Influenza-like Illness Surveillance Network, where outpatient visits are monitored for influenza-like illness defined as fever plus cough or sore throat, can include reports due to infection with cocirculating respiratory pathogens that may be clinically indistinguishable from influenza, including COVID-19 and RSV. In addition, changes in health care–seeking behavior due to the COVID-19 pandemic, both in terms of provider choice and timing relative to illness onset and severity, will be important to consider when assessing surveillance data collected from health care settings. Additionally, the expansion of remote or telehealth options will complicate surveillance, particularly for laboratory-based surveillance. The Influenza Division will continue to leverage the strengths of different surveillance systems to provide a comprehensive understanding of seasonal influenza virus activity. In addition, in order to better understand the burden of influenza in relation to other respiratory viruses like RSV and SARS-CoV-2, the Influenza Division has cosupported the development of multipathogen respiratory virus surveillance networks and developed publicly facing summaries that compare acute respiratory illness emergency department visits for laboratory-confirmed influenza, RSV, and COVID-19 [14] and weekly in-season rates of laboratory-confirmed influenza, COVID-19, and RSV-associated hospitalizations by age, sex, and race or ethnicity [15]. Influenza surveillance and communication efforts will continue to adapt in the uncertain and changing landscape of continued respiratory virus cocirculation.

## Estimating Influenza Disease Incidence Using Analytic Methods That Account for the Underdetection of Influenza Provides a More Complete Picture of the Burden and Severity of the Influenza Season

Not all people seek medical care for respiratory illnesses, and not all of those who do will be tested to confirm infection with a specific pathogen. Even when an individual seeks medical care and is tested, this may not occur during the time window of their illness when a collected sample has the potential to test positive (ie, the pathogen or antibodies may no longer be detectable). A variety of viral and bacterial respiratory illnesses are clinically indistinguishable from one another, complicating diagnosis based on symptoms or clinical findings alone. Laboratory testing for specific pathogens can be more or less common in certain care settings, for different age groups, and at differing levels of disease severity. To account for the incomplete detection of influenza virus infection from clinical testing in health care settings, models used to estimate the disease burden incorporate information on health care usage and laboratory testing for respiratory viruses [16]. Each year, the CDC uses mathematical and statistical models to estimate the broader disease burden from influenza in the US population; since 2018, this has been estimated weekly during the influenza season [17]. In the 2022-2023 season, the first preliminary in-season weekly influenza burden estimates were released in October [4], earlier than any other season due to the atypically high levels of influenza activity observed at that time of year.

The ongoing effects of the COVID-19 pandemic on health care usage and testing for respiratory viruses [18], including influenza, are unclear and will likely continue to evolve over time. Major changes to health care–seeking behaviors, including where patients are seeking care and the use of clinical versus at-home diagnostic testing, could influence trends observed in long-standing surveillance systems, impact ongoing efforts to forecast influenza activity, and introduce new biases into epidemiological and clinical studies that rely on the results of clinical testing. The CDC will continue to make estimates using the best available data, adapt to challenges presented by atypical seasonality, and assess developments in care-seeking behaviors and influenza testing [19]. Communicating in-season and end-of-season burden estimates through the CDC website and FluView reports will help promote public awareness and add to our understanding of influenza burden alongside COVID-19 burden. These ongoing activities will ensure that influenza burden estimation continues to be improved, using the best available statistical methods and data sources.

## Collaborative Forecasting Efforts Hosted by the CDC Use Surveillance Data to Look Ahead and Provide Quantitative Forecasts for Short-Term Trends in Influenza Activity

Although these traditional surveillance systems and disease burden estimates are comprehensive, they measure influenza activity after it has occurred, which limits their utility to anticipate future trends and inform risk assessment, resource allocation, and health care preparedness. To address these limitations, the CDC has been collaborating with and supporting public health officials and researchers from academia, industry, and the government in open forecasting challenges since the 2013-2014 season [20]. This process has ensured that forecasting targets are relevant to public health, forecast data are openly available and communicated; and the evaluation of forecast performance across different targets, seasons, geographic locations, and methods is transparent [21-23]. This foundation helped lead to the rapid establishment of the COVID-19 forecast hub in early 2020 [24-26].

Building on 10 years of experience and working in collaboration with academic and industry partners, including 2 Centers of Excellence in Influenza Forecasting (the University of Massachusetts and Carnegie Mellon University), the CDC has regularly communicated short-term forecasts at the national, state, and territory level for the 2023-2024 influenza season for the weekly number of patients hospitalized with laboratory-confirmed influenza based on data from the National Healthcare Safety Network (NHSN) hospital admission system (previously known as the US Department of Health & Human Services (HHS) system HHS-Protect) [13,27]. This hospitalization-based forecast target was adopted during the 2021-2022 season when reporting of laboratory-confirmed influenza hospital admissions became mandatory in February 2022 at the state- and hospital facility- level and is different from the forecast target used before the COVID-19 pandemic, which was based on outpatient visits for influenza-like illness [20]. This shift was made because of changes in outpatient care seeking associated with the pandemic, the continued cocirculation of SARS-CoV-2 and RSV, which complicated the interpretation of influenza-like illness data, and the new availability of laboratory-confirmed influenza hospital admission data through NHSN for all 50 states. This centralized source of virus-specific hospitalization data may provide actionable and reliable information moving forward [28,29]. Experience from using the NHSN hospital admission indicator for influenza forecasting during the 2021-2022 and 2022-2023 influenza seasons [30], as well as during other forecasting and modeling efforts during the COVID-19 pandemic, indicates that the NHSN hospital admission data [13], which became available during the COVID-19 pandemic, are a robust source of timely information about the impact of influenza in US hospitals. Reported data include daily state-level counts of total hospitalized patients, previous day’s admissions, and total hospitalized intensive care unit patients each with a laboratory-confirmed influenza virus infection [13].

Insights from past forecasting efforts, including for the COVID-19 pandemic and past influenza seasons, can provide users of forecasts with valuable takeaways for interpreting influenza forecasts with appropriate levels of caution. First, forecasts are less reliable during periods of rapid change (eg, during periods of sharp increases in incidence or when activity is peaking). During these periods, forecasts are often unable to reliably predict changing trends, and interim evaluation of early season forecasts received during the periods of the 2022-2023 season when influenza activity was increasing rapidly or peaking indicated that more reported hospitalizations than expected fell outside of the forecast prediction intervals, indicating a lack of forecast reliability. Improvements in forecasting methodology are needed to ensure that periods of rapid change can be forecasted reliably. Due to the unreliability of forecasts observed in these periods, the Influenza Division did not start public communication of these forecasts until observed performance became more reliable (January 2023). To begin addressing this limitation, the FluSight challenge [27] piloted a set of new forecast targets for probabilities of observing categorical changes in hospital admission trends, such as increases and decreases above certain thresholds, at state, territory, and national levels and will continue the use of these targets in the 2023-2024 season. Second, ensemble models typically outperform individual model forecasts, making them a more robust choice for forecasts with dependably accurate performance [22-24,26]. Finally, forecast performance degrades as they predict farther into the future, as evidenced by the higher performance of 1-week ahead forecasts than 2-, 3-, and 4-week ahead forecasts [22], and the CDC may also pause communication of these longer-horizon forecasts in periods of rapid change. In addition to the challenges associated with rapid increases, atypical seasonality, and new data sources for forecasting present additional challenges for forecasting influenza during and following the COVID-19 pandemic since many forecasting models rely on historical data to inform predictions of seasonal trends. Ongoing discussions with academic and industry partners that complement collaborations with the Council for State and Territorial Epidemiologists, including the pilot activities mentioned above, will help ensure that forecasting and modeling results are shared, discussed, and interpreted in a timely manner.

## Additional Modeling Approaches Are Needed to Increase Our Understanding of the Potential Severity and Timing of the Upcoming Influenza Seasons

Inherent uncertainties in epidemiological conditions and the effects of interventions over longer time horizons require different modeling frameworks than forecasting alone. Therefore, the CDC started collaborating with the Scenario Modeling Hub to use a multiple-model approach to consider potential courses of the 2022-2023 influenza season, including 4 scenarios covering higher and lower rates of vaccine impact and different levels of preexisting immunity [31-33]. The simultaneous consideration of multiple scenarios (sets of assumptions) allows modelers to consider what could happen under a variety of different circumstances. Results from the first round of the influenza Scenario Modeling Hub suggested in September 2022 that hospitalizations in the 2022-2023 influenza season would be higher than in recent seasons [32]. Early in the 2022-2023 season, projections for scenarios with lower population-level immunity demonstrated the plausibility of peak hospitalizations comparable to those of larger prepandemic influenza seasons, while projections for scenarios with higher vaccine effectiveness and coverage were accompanied by substantial reductions in peak size and cumulative burden of hospitalizations due to influenza [32]. Subsequent rounds of the Influenza Scenario Modeling Hub undertaken in November and December 2022 reinforced these findings that projected peak hospitalizations could be comparable to those of larger prepandemic influenza seasons in the most plausible scenarios [32]. As demonstrated by several completed rounds of the COVID-19 Scenario Modeling Hub [34], these longer-term projections provide a useful information source for intervention planning, preparedness, and decision-making [35-38]. By continuing to engage with the Scenario Modeling Hub in weekly meetings, the CDC will be able to provide insight on which questions are of most interest for public health practice and preparedness during the 2023-2024 influenza season to help guide scenario development as well as lend expertise on data sets that may enhance modeling efforts. Results from the Influenza Scenario Modeling Hub may be used to inform potential peak timing and intensity. Additionally, influenza modelers within the CDC will continue to engage with the broader infectious disease modeling community. Information on changing influenza epidemiology and surveillance systems will be regularly communicated across forecasting groups, modeling groups, and data curators. This will be achieved in part through coordinated efforts with partners in the Models of Infectious Disease Agent Study Network [39].

## Conclusion

The COVID-19 pandemic has changed many things that could influence trends observed in long-standing influenza surveillance systems. These changes have impacted ongoing efforts to describe influenza disease burden and forecast influenza activity and introduced new biases into epidemiologic and clinical studies that rely on the results of clinical testing. However, the pandemic has also provided opportunities for innovation that could better inform and support influenza efforts moving forward, including novel data sources such as the NHSN hospital admission data set, the ability to compare the impact of multiple respiratory pathogens in different clinical settings, and increased engagement with the public and external partners. Prepandemic approaches used to inform influenza prevention and control efforts, such as surveillance, burden estimation, and modeling, will continue to be reevaluated to ensure their reliability. State-level data may be used in the future to provide more spatially resolved estimates of burden beyond the national burden estimates that are currently provided. The US CDC Influenza Division remains committed to these key data and analytic activities, including the simultaneous monitoring of syndromic influenza surveillance with complimentary data sources, as well as providing a central platform for collaboration and communication across disciplines and institutions to ensure that information to inform influenza prevention, situational awareness, and control remains robust and readily available.

## Abbreviations

CDC: Centers for Disease Control and Prevention
HHS: Health & Human Services
NHSN: National Healthcare Safety Network
RSV: respiratory syncytial virus
